# Flow Cytometric Profiling Reveals Platelet Dysfunction and Impaired Immune Communication in Acute and Chronic Cerebrovascular Disease

**DOI:** 10.1002/jcla.70192

**Published:** 2026-03-09

**Authors:** Chih‐Lung Shen, Li‐Yu Huang, Jhong‐Kuei Chen, Sheng‐Tzung Tsai, Yi‐feng Wu

**Affiliations:** ^1^ Division of Hematology‐Oncology, Department of Internal Medicine Hualien Tzu Chi Hospital, Buddhist Tzu Chi Medical Foundation Hualien Taiwan; ^2^ Department of Chinese Medicine Hualien Tzu Chi Hospital, Buddhist Tzu Chi Medical Foundation Hualien Taiwan; ^3^ Integration Center of Traditional Chinese and Modern Medicine Hualien Tzu Chi Hospital, Buddhist Tzu Chi Medical Foundation Hualien Taiwan; ^4^ School of Post‐Baccalaureate Chinese Medicine Tzu Chi University Hualien Taiwan; ^5^ Department of Neurosurgery Hualien Tzu Chi Hospital, Buddhist Tzu Chi Medical Foundation Hualien Taiwan; ^6^ School of Medicine Tzu Chi University Hualien Taiwan

**Keywords:** blood biomarkers, chronic cerebrovascular diseases, intracerebral hemorrhage, platelet function, P‐selectin

## Abstract

**Introduction:**

Beyond hemostasis and thrombosis, platelets are increasingly recognized as multifunctional cells involved in inflammation, vascular remodeling, and neurological diseases. Chronic cerebrovascular diseases impair cerebral blood flow and oxygen supply, leading to cognitive and motor dysfunction, where platelets may act as peripheral sensors reflecting neurovascular stress. This study investigated platelet activation profiles and platelet–leukocyte interactions in acute intracerebral hemorrhage (aICH), subacute intracerebral hemorrhage (sICH), and chronic cerebrovascular diseases (CCD).

**Methods:**

Flow cytometry was used to assess platelet functions, including PAC‐1 (activated GPIIb/IIIa) and P‐selectin (CD62P), and platelet–leukocyte aggregates, including myeloid‐platelet, lymphocyte‐platelet, and monocyte‐platelet in 20 CCD, 20 aICH, 30 sICH patients, and 48 healthy controls.

**Results:**

PAC‐1 expression was significantly reduced in aICH and sICH but elevated in CCD, indicating impaired integrin activation in acute and subacute stages and compensatory hyperreactivity in chronic disease. P‐selectin expression was markedly decreased in CCD under both ADP and TRAP stimulation, suggesting persistent α‐granule dysfunction in chronic conditions. Lymphocyte–platelet aggregates, especially T‐cell interactions, were consistently diminished across all patient groups, reflecting disturbed platelet–immune communication with T lymphocytes.

**Conclusion:**

Patients with cerebrovascular disorders show distinct platelet activation patterns and impaired immune crosstalk. Reduced P‐selectin expression and decreased lymphocyte–platelet aggregation, especially T‐cell, indicate sustained platelet dysfunction and disrupted neuroinflammatory regulation. Flow cytometric assessment of these parameters provides valuable insight into neurovascular diseases at different stages and potential biomarkers for disease monitoring.

## Graphical abstract

Platelets act as sensors linking inflammation and neurovascular health. Flow cytometry revealed reduced PAC‐1 in acute/subacute ICH but elevated in chronic disease, while P‐selectin remained suppressed. T lymphocyte–platelet aggregates declined across groups, indicating disrupted immune crosstalk, and these biomarkers reflect stage‐specific platelet dysfunction in brain injury.

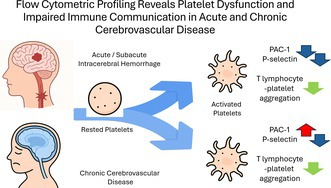

## 1 Introduction

Beyond hemostasis and thrombosis, platelets and their organelles are involved in the pathophysiology of diabetes, cancer, cardiovascular, and neurological diseases [1]. Chronic cerebrovascular diseases are conditions that affect the blood vessels in the brain, leading to reduced blood flow and oxygen supply, including moyamoya disease, intracranial atherosclerotic disease, and cerebral small vessel disease. Chronic cerebrovascular diseases can cause symptoms such as headache, cognitive impairment, and motor dysfunction [2]. Increasing evidence shows that platelets respond to diverse stressors, providing a unique perspective on their role as systemic sensors in human diseases. Platelets have been proposed as peripheral probes that reflect neuronal dysfunction and neuroinflammatory processes [3]. In cerebrovascular diseases, platelets play a dual role: they promote microthrombosis and ischemia through aggregation and adhesion, and modulate neuroinflammation and endothelial function by releasing cytokines, chemokines, and growth factors. Based on a previous study, activated platelets can orchestrate vascular inflammation by releasing CCN1/CYR61, which promotes the recruitment and endothelial patrolling of Ly6Clow monocytes and facilitates subsequent neutrophil recruitment [4].

Neuroimmune interactions are crucial for maintaining brain homeostasis and plasticity throughout life. Platelets have gained recognition for their roles beyond hemostasis, particularly in mediating blood–brain communication [5]. They participate in inflammation, vascular remodeling, and tissue repair through the release of bioactive molecules stored in α‐granules. This dynamic turnover allows platelets to sense and adapt to environmental changes, linking systemic alterations to neural function and vascular health.

Platelets therefore play crucial roles not only in hemostasis but also in inflammation, neurovascular injury, and tissue repair. Platelets are also emerging as key players in regenerative medicine. Following tissue injury and inflammation, they release growth factors such as platelet‐derived growth factor, platelet factor 4, and vascular endothelial growth factor, promoting tissue remodeling and regeneration, including within the central nervous system (CNS) [6, 7]. α‐Granules additionally contain bioactive mediators that support neurogenesis independently of secretion [8, 9, 10]. Beyond repair, platelets also respond to vascular and neuroinflammatory signals. Administration of brain‐derived gangliosides in animal models induces massive platelet activation and degranulation, mediated through P‐selectin (CD62P), emphasizing their role in neuroimmune crosstalk [11]. Previous studies have demonstrated that platelet activation markers such as GPIIb/IIIa and P‐selectin reflect platelet reactivity and their capacity to form platelet–leukocyte aggregates, which are implicated in secondary brain injury and recovery [12, 13, 14]. Platelet–leukocyte aggregates represent a dynamic interface between hemostasis and inflammation. The crosstalk between activated platelets and circulating leukocytes amplifies inflammatory cascades, endothelial activation, and microvascular dysfunction—processes highly relevant to brain injury and neurodegeneration [15]. In the neurologic field, it serves as both a pathophysiologic mechanism linking thrombosis and inflammation, and a potential biomarker for disease progression and therapeutic response. GPIIb/IIIa and P‐selectin represent distinct but complementary indicators of platelet activation, reflecting different stages of the activation cascade. PAC‐1 is a conformation‐specific antibody that binds to the activated form of the integrin αIIbβ3 (GPIIb/IIIa), which mediates fibrinogen binding and platelet aggregation. Thus, PAC‐1 positivity indicates early platelet activation, reflecting the functional readiness for aggregation. In contrast, P‐selectin (CD62P) is a marker of α‐granule secretion, translocating from the granule membrane to the platelet surface upon degranulation. Its expression denotes late activation events associated with granule release, platelet–leukocyte interactions, and inflammatory signaling [16, 17, 18].

According to the International Society on Thrombosis and Haemostasis, platelet function in acquired disorders can be evaluated by flow cytometry using glycoprotein IIb/IIIa (PAC‐1 binding) and P‐selectin (CD62P) expression, as well as leukocyte–platelet aggregates [19]. This technique offers high sensitivity and minimal sample requirements, allowing accurate assessment even at low platelet counts. In pathological conditions such as intracerebral hemorrhage (ICH) or other chronic cerebrovascular diseases, the differential expression of these markers may reveal distinct alterations in platelet function [20]. Decreased PAC‐1 expression may suggest impaired activation of the fibrinogen receptor, whereas sustained or reduced P‐selectin expression may indicate dysregulated secretion pathways or chronic platelet exhaustion. However, the evolutionary changes of different biomarkers of platelet function at different stages of cerebrovascular incidence were not explored before.

This study aimed to investigate platelet activation profiles, including glycoprotein IIb/IIIa and P‐selectin expression, and platelet–leukocyte interactions in patients with acute intracerebral hemorrhage (aICH), subacute intracerebral hemorrhage (sICH), and chronic cerebrovascular diseases (CCD) compared with healthy controls.

## 2 Materials and Methods

Between January 2023 and August 2025, peripheral blood samples were collected from aICH patients, sICH patients, CCD patients, and healthy volunteers at the Hualien Tzu Chi Hospital, Taiwan.

Patients were enrolled into four groups. The CCD group comprised clinically stable patients with chronic intracranial lesions identified by medical history and confirmed by computed tomography (CT) or magnetic resonance imaging (MRI); all were regularly followed at the neurosurgery outpatient clinic and were not receiving antiplatelet, anticoagulant, or hemostatic medications at enrollment, and blood samples were collected during routine outpatient follow‐up visits. The aICH group included patients with traumatic acute intracranial hemorrhage who were sampled within 24 h of the event, prior to any transfusion or surgical intervention. The sICH group included patients with traumatic intracranial hemorrhage who were sampled 7–14 days after the initial event; all were clinically stable in the general ward and had not received transfusions or hemostatic agents before sampling. A total of 48 healthy controls were also enrolled for comparison.

This study was approved by the Institutional Review Board of Tzu Chi General Hospital (IRB112‐027‐B, IRB109‐049‐B and IRB108‐200‐B). Informed consent was obtained after explaining the study protocol.

Routine hematologic and coagulation parameters were measured, and platelet function was assessed by flow cytometry as below.

### 2.1 Platelet Surface Markers of Activated Platelets

As previously described, platelet activation was also assessed using whole‐blood flow cytometry [21]. Briefly, citrate‐anticoagulated whole blood was incubated with an antibody cocktail containing fluorescein isothiocyanate (FITC)‐conjugated PAC‐1, phycoerythrin (PE)‐conjugated anti‐P‐selectin, and PE‐Cy5–conjugated anti‐CD42b, with or without 20 μM ADP or thrombin receptor–activating peptide (TRAP) for 15 min at room temperature. Samples were then fixed with 1% formaldehyde. All antibodies were obtained from BD Pharmingen (Franklin Lakes, NJ, USA) [22].

Positivity thresholds were defined using matched isotype controls, with gates set to include ≤ 1% of negative‐control events and then uniformly applied across all samples and stimulation conditions. For platelet activation analysis (PAC‐1 and P‐selectin), platelets were first identified on a logarithmic FSC versus SSC plot as the population of small, low–side scatter events, and this gate was refined by selecting platelet‐marker–positive events (CD42b+) to exclude debris, red cell fragments, and microparticles. PAC‐1 and P‐selectin expression were then quantified within the gated platelet population using bivariate plots (e.g., PAC‐1 vs. CD62P), with quadrant boundaries established from unstimulated controls to define PAC‐1 positivity (activated GPIIb/IIIa/αIIbβ3 conformation), P‐selectin positivity (α‐granule secretion), and dual‐positive events representing fully activated platelets.

### 2.2 Platelet–Leukocyte Aggregation

We have clarified the antibody panels and gating strategy used for platelet–leukocyte aggregate analysis. The gating strategy was designed to identify platelet aggregates associated with specific leukocyte subtypes of interest. CD33 was used to define the myeloid lineage (including neutrophils), and CD14 was used to identify monocytes. In addition, CD3 and CD19 were used to identify T and B lymphocytes, respectively, for the evaluation of lymphocyte–platelet aggregates. Platelet–leukocyte aggregates were identified by CD42b positivity within each leukocyte subset, and the percentage of aggregates was calculated as the proportion of CD42b‐positive events within the parent leukocyte gate (i.e., CD42b+ events of a given leukocyte subset divided by total events of the same subset).

Briefly, 100 μL of whole blood was incubated at room temperature with an antibody cocktail containing FITC‐conjugated anti‐CD14, PE‐conjugated anti‐CD33, PerCP‐conjugated anti‐CD45, and APC‐conjugated anti‐CD42b, followed by red blood cell lysis using FACS Lysing Solution (Becton Dickinson, BD Biosciences, Oxford, UK). After centrifugation at 400× *g* for 5 min, the cell pellet was resuspended in 500 μL of HEPES‐buffered saline solution and analyzed by flow cytometry. All antibodies were obtained from BD Pharmingen (Franklin Lakes, NJ, USA) [22, 23].

The gating strategy was designed to identify platelet–leukocyte aggregates associated with specific leukocyte subtypes. Within the CD45+ singlet population, myeloid cells were defined by CD33 positivity, and monocytes were identified as CD14‐positive events. T and B lymphocytes were further identified within the lymphocyte gate as CD3‐positive and CD19‐positive populations, respectively. Platelet–leukocyte aggregates were defined as CD42b‐positive events within each leukocyte subset and were quantified as the percentage of CD42b+ events divided by total events in the corresponding parent leukocyte gate. The detailed gating strategy is shown in Figures S1 and S2.

### 2.3 Flow Cytometry

Flow cytometric analysis of platelet surface antigens was performed using a FACSCalibur flow cytometer (Becton Dickinson Immunocytometry Systems, San Jose, CA, USA). Data were analyzed with the WinList software (Verity Software, Topsham, ME, USA).

### 2.4 Statistical Analysis

The collected data were entered into GraphPad Prism, version 8 (GraphPad Software, San Diego, CA, USA), for analysis. Laboratory parameters, platelet surface antigen expression, platelet–leukocyte aggregation, and cytokine levels at different time points were compared using one‐way ANOVA. Data are presented as means with 95% confidence intervals (95% CIs). A *p*‐value < 0.05 was considered statistically significant.

## 3 Results

### 3.1 Demographic and Laboratory Characteristics

We enrolled 20 patients with acute intracerebral hemorrhage (aICH), 30 with subacute intracerebral hemorrhage (sICH), 20 patients with chronic cerebrovascular diseases (CCD), and 48 healthy controls.

The sex distribution differed across groups (Table 1). Pairwise comparisons versus the control group using Fisher's exact test showed that the aICH, sICH, and CCD groups had significantly different sex distributions compared with controls (*p* < 0.01). This result was mainly driven by the markedly imbalanced male‐to‐female ratio in the control group, which influenced the *p* values.

**TABLE 1:** The demographic and laboratory characteristics.

	Healthy control	aICH	sICH	CCD	*p*		
					aICH vs. control	sICH vs. control	CCD vs. control
Numbers	48	20	30	20			
Male:female	8:40	12:8	19:11	10:10	< 0.01**	< 0.01**	< 0.01**
Age	41.8 ± 10.0	62.4 ± 19.4	62.7 ± 18.8	74.9 ± 5.5	< 0.01**	< 0.01**	< 0.01**
White blood count (WBC/μL)	6695 ± 1727	11,310 ± 5482	8460 ± 3397	6630 ± 1769	< 0.01**↑	0.01**↑	0.88
Hemoglobulin (g/dL)	13.0 ± 1.3	10.9 ± 2.8	11.6 ± 2.0	12.9 ± 1.8	< 0.01**↓	< 0.01**↓	0.86
Platelet (×1000/μL)	278.5 ± 63.6	205.1 ± 80.6	244.8 ± 105.9	264.8 ± 77.8	< 0.01**↓	< 0.01**↓	0.51
PT (s)	10.1 ± 0.5	11.7 ± 1.7	10.9 ± 0.9	10.9 ± 0.4	All in normal range		
aPTT (s)	28.8 ± 3.0	26.6 ± 3.0	26.8 ± 3.9	16.2 ± 1.7	All in normal range		
Neutrophil (%)	60.8 ± 9.4	77.2 ± 13.7	69.5 ± 14.6	66.5 ± 9.7	< 0.01**↑	< 0.01**↑	0.05
Lymphocyte (%)	30.5 ± 7.9	15.4 ± 12.0	20.3 ± 11.9	24.8 ± 8.9	< 0.01**↓	< 0.01**↓	0.02*↓
Monocyte (%)	5.7 ± 1.9	5.2 ± 1.7	6.2 ± 2.1	5.8 ± 1.0	0.53	0.27	0.19

*Note:* Mean ± SD; *p*‐value of gender by Fisher's exact test; *p*‐value of other results by Mann–Whitney test, non‐parametric test.

Abbreviations: aICH, acute intracerebral hemorrhage; CCD, chronic cerebrovascular disease; sICH, subacute intracerebral hemorrhage.

* *p* < 0.05.

** *p* < 0.01.

In CCD groups, based on medical history and neuroimaging findings, age‐related brain atrophy was identified in 8 patients, small vessel ischemic disease in 6 patients, old infarction with brain tissue loss in 4 patients, and chronic subdural hematoma in 2 patients. All patients had brain lesions confirmed by computed tomography (CT) or magnetic resonance imaging (MRI) and were regularly followed up at the neurosurgery outpatient clinic, remaining clinically stable without the use of antiplatelet, anticoagulant, or hemostatic medications. Blood samples were collected during routine follow‐up visits in the outpatient department.

In aICH group, 20 patients were presented with traumatic acute intracranial hemorrhage within 24 h. Blood samples were collected before any transfusion or surgical intervention. In the sICH group, blood samples from 30 patients were obtained 7 to 14 days after the initial traumatic intracranial hemorrhage. All patients were clinically stable in general ward and did not receive transfusions or hemostatic agents. A total of 48 health controls were also enrolled for comparison.

The mean ages of the aICH (62.4 ± 19.4 years), sICH (62.7 ± 18.8 years), and CCD (74.9 ± 5.5 years) groups were significantly higher than those of the control group (41.8 ± 10.0 years; *p* < 0.01 for all comparisons). The detailed data and sex distribution were shown in Table 1.

### 3.2 Hematology and Coagulation Profiles

Compared with controls, white blood cell counts were elevated in aICH (11,310 ± 5482/μL, *p* < 0.01) and sICH (8460 ± 3397/μL, *p* = 0.01), but not in CCD (6630 ± 1769/μL, *p* = 0.88). Hemoglobin was lower in aICH (10.9 ± 2.8 g/dL) and sICH (11.6 ± 2.0 g/dL) versus controls (13.0 ± 1.3 g/dL; both *p* < 0.01), and similar in CCD (12.9 ± 1.8 g/dL, *p* = 0.86). Platelet counts were reduced in aICH (205.1 ± 80.6 × 10^3^/μL) and sICH (244.8 ± 105.9 × 10^3^/μL) relative to controls (278.5 ± 63.6 × 10^3^/μL; both *p* < 0.01), with no difference for CCD (264.8 ± 77.8 × 10^3^/μL, *p* = 0.51).

**FIGURE 1:**
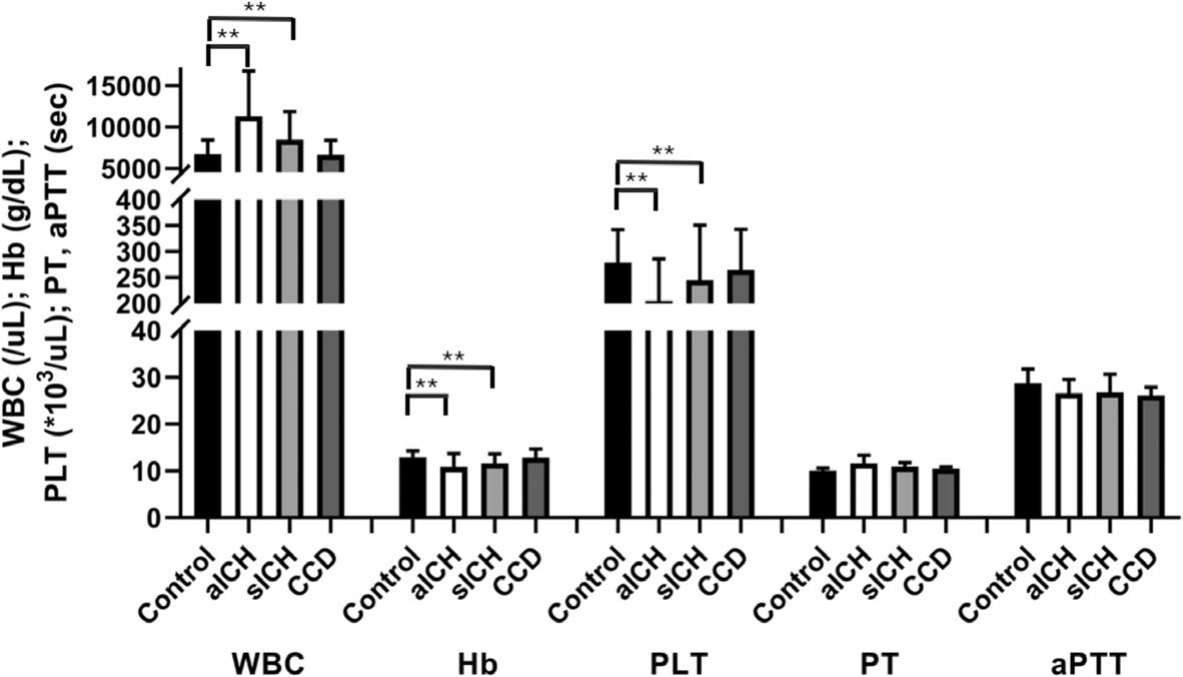
Hematology and coagulation profiles in patients with cerebrovascular diseases. White blood cell (WBC), hemoglobin (Hb), and platelet (PLT) counts, as well as prothrombin time (PT) and activated partial thromboplastin time (aPTT), were compared among healthy controls, patients with acute intracerebral hemorrhage (aICH), subacute intracerebral hemorrhage (sICH), and chronic cerebrovascular disease (CCD). Detailed numerical values are summarized in Table 1. **p < 0.01.

Prothrombin time was mildly prolonged across patient groups (aICH 11.7 ± 1.7 s; sICH 10.9 ± 0.9 s; and CCD 10.9 ± 0.4 s). Activated partial thromboplastin time was shorter in CCD (16.2 ± 1.7 s, *p* = 0.01) and showed a non‐significant trend toward shorter times in aICH (26.6 ± 3.0 s, *p* = 0.07) and sICH (26.8 ± 3.9 s, *p* = 0.08) versus controls (28.8 ± 3.0 s). But prothrombin time and activated partial thromboplastin time were in the normal range in all groups. The detailed data are shown in Table 1 and Figure 1.

### 3.3 Platelet Integrin Activation (PAC‐1 Binding to Activated GPIIb/IIIa)

**FIGURE 2:**
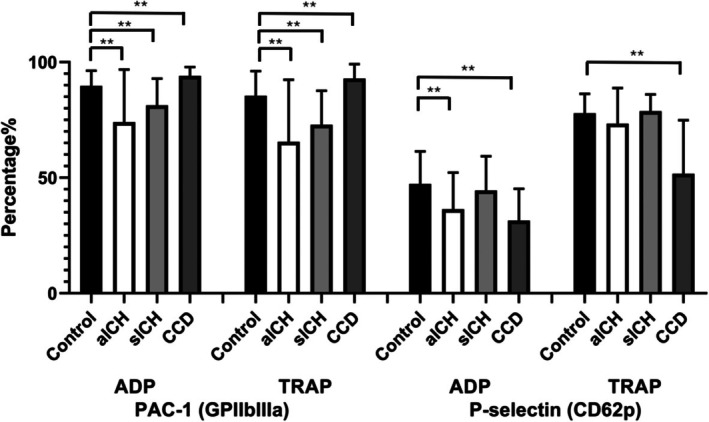
Platelet surface activation profiles across disease states. Percentages of platelet surface expression of PAC‐1 (GPIIb/IIIa) and P‐selectin (CD62P) under stimulation with ADP or thrombin receptor–activating peptide (TRAP) in healthy controls, patients with acute intracerebral hemorrhage (aICH), subacute intracerebral hemorrhage (sICH), and chronic cerebrovascular disease (CCD). **p < 0.01. Detailed numerical values are summarized in Table 2.

Across all agonist conditions, PAC‐1 positivity was increased in CCD groups and depressed in aICH and sICH groups compared with controls. Under ADP stimulation, PAC‐1 expression increased to 94.1% ± 3.8% in CCD but decreased to 74.1% ± 22.7% in aICH and 81.3% ± 11.6% in sICH, compared with healthy controls (89.7% ± 6.6%; *p* ≤ 0.01 for all). Following TRAP stimulation, PAC‐1 expression increased to 92.9% ± 6.2% in CCD but decreased to 65.6% ± 26.8% in aICH and 72.9% ± 14.7% in sICH, compared with healthy controls (85.5% ± 10.6%; *p* ≤ 0.01 for all). The detailed data are shown in Table 2 and Figure 2.

**TABLE 2:** Total study results including expression of GPIIbIIIa (PAC‐1) and p‐selectin in human platelets.

	Healthy control (*N* = 48)	aICH (*N* = 20)	sICH (*N* = 30)	CCD (*N* = 20)	*p*		
					aICH vs. control	sICH vs. control	CCD vs. control
PAC‐1 (activated GPIIb/IIIa) percentage (%)							
Unstimulated	1.72 ± 1.19	2.90 ± 2.10	2.91 ± 3.73	2.67 ± 1.42	< 0.02*↑	< 0.01**↑	0.04*↑
ADP stimulation	89.7 ± 6.6	74.1 ± 22.7	81.3 ± 11.6	94.1 ± 3.8	< 0.01**↓	< 0.01**↓	< 0.01**↑
TRAP stimulation	85.5 ± 10.6	65.6 ± 26.8	72.9 ± 14.7	92.9 ± 6.2	< 0.01**↓	< 0.01**↓	< 0.01**↑
P‐selectin (CD62p) percentage (%)							
Unstimulated	1.84 ± 1.32	1.60 ± 1.35	3.21 ± 3.66	2.48 ± 2.77	0.47	0.02*↑	0.21
ADP stimulation	47.3 ± 14.1	36.4 ± 15.9	44.4 ± 14.9	31.5 ± 13.7	< 0.01**↓	0.38	< 0.01**↓
TRAP stimulation	77.9 ± 8.4	73.3 ± 15.4	78.8 ± 7.3	51.7 ± 23.2	0.36	0.68	< 0.01**↓

*Note:* Mean ± SD; *p*‐value by Mann–Whitney test, non‐parametric test.

Abbreviations: aICH, acute intracerebral hemorrhage; CCD, chronic cerebrovascular disease; sICH, subacute intracerebral hemorrhage.

* *p* < 0.05.

** *p* < 0.01.

Based on the above results, PAC‐1 expression was decreased during the initial phase of intracranial hemorrhage and remained reduced even in the subacute phase after the event. In contrast, patients with chronic cerebrovascular diseases evaluated in the outpatient department showed higher PAC‐1 expression compared with healthy controls. The deficits were most pronounced in the aICH group under submaximal stimulation, indicating impaired inside‐out integrin activation, particularly in the acute phase.

### 3.4 Platelet α‐Granule Secretion (P‐Selectin/CD62p Expression)

Under unstimulated conditions, PAC‐1 expression was slightly higher in the CCD (2.90 ± 2.10), aICH (2.91 ± 3.73), and sICH (2.67 ± 1.42) groups than in healthy controls (1.72 ± 1.19; *p* ≤ 0.05 for all comparisons). However, absolute values in all groups remained below 3%, indicating a very small magnitude of baseline activation; thus, despite statistical significance, the difference is unlikely to be clinically meaningful. With ADP stimulation, P‐selectin expression decreased to 36.4% ± 15.9% in aICH, 44.4% ± 14.9% in sICH, 31.5% ± 13.7% in CCD, and 47.3% ± 14.1% in controls (aICH, *p* < 0.01; sICH, *p* = 0.38; CCD, *p* < 0.01). Following TRAP stimulation, P‐selectin expression decreased to 73.3% ± 15.4% in aICH, 78.8% ± 7.3% in sICH, 51.7% ± 23.2% in CCD, and 77.9% ± 8.4% in controls (aICH, *p* = 0.36; sICH, *p* = 0.68; CCD, *p* < 0.01). However, P‐selectin expression showed no significant differences in sICH under ADP stimulation, or in aICH and sICH under TRAP stimulation. The detailed data are shown in Table 2 and Figure 2.

Under unstimulated conditions, there were almost no differences in P‐selectin among the four groups. This is expected because, in resting platelets, P‐selectin is not expressed on the platelet surface unless the platelets are activated. P‐selectin secretion capacity was significantly reduced in the CCD group under ADP or TRAP stimulation, whereas the aICH and sICH groups approached control levels when exposed to strong stimulation. Reduced P‐selectin expression without evidence of recovery was still observed in the CCD group, even under chronic brain conditions.

### 3.5 Platelet–Leukocyte Aggregates (PLAs)

Myeloid–platelet and monocyte–platelet aggregates were comparable in sICH and CCD versus controls (all *p* ≥ 0.11), but myeloid–platelet aggregates were reduced in aICH (21.1% ± 11.2% vs. 28.4% ± 8.1%, *p* < 0.01). Lymphocyte–platelet aggregates were consistently decreased across aICH (11.3% ± 4.7%), sICH (13.5% ± 5.8%), and CCD (13.9% ± 5.3%) compared with controls (23.2% ± 6.1%; all *p* < 0.01).

In lymphocyte subsets and their PLAs, circulating T‐cell percentages were higher in sICH (87.0% ± 6.2%) and CCD (88.5% ± 4.8%) than controls (83.7% ± 4.2%; both *p* < 0.01), whereas B‐cell percentages were correspondingly lower (sICH 13.0% ± 6.2% and CCD 11.5% ± 4.8% vs. controls 16.3% ± 4.2%; both *p* < 0.01). aICH did not differ from controls in T‐ or B‐cell proportions.

**FIGURE 3:**
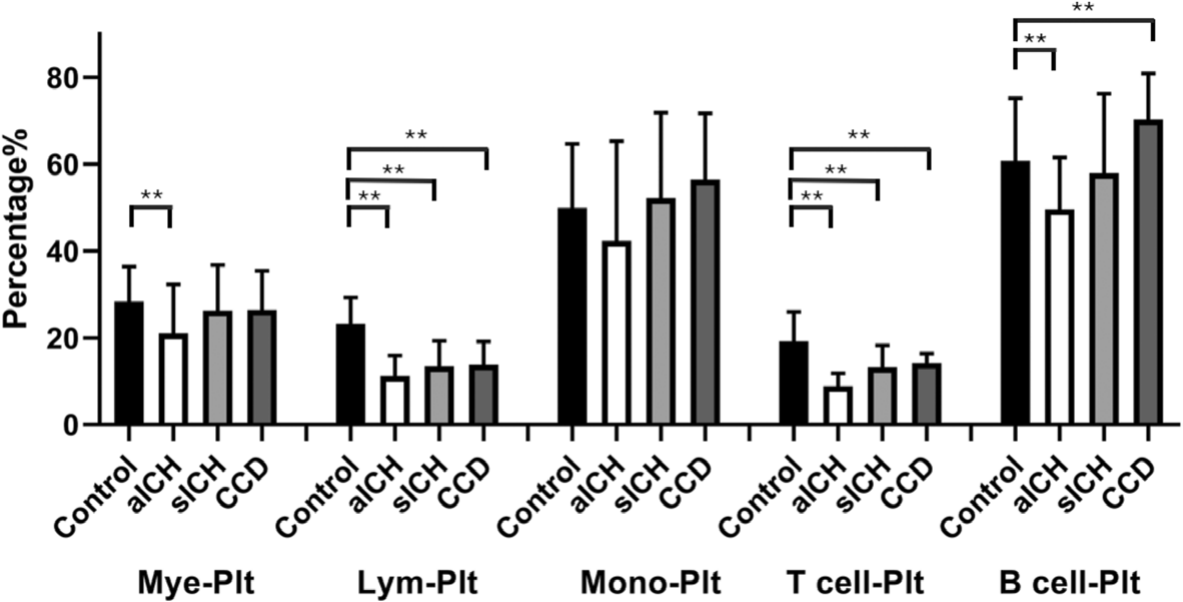
Platelet–leukocyte aggregation. Quantitative analysis of platelet–leukocyte aggregates, including myeloid–platelet (Mye–Plt), lymphocyte–platelet (Lym–Plt), monocyte–platelet (Mono–Plt), T lymphocyte–platelet (T cell–Plt), and B lymphocyte–platelet (B cell–Plt) interactions. **p < 0.01. Detailed numerical values are summarized in Table 3.

Functionally, T‐cell–platelet aggregates were significantly reduced in all patient groups (aICH 8.9% ± 2.9%, sICH 13.2% ± 5.1%, and CCD 14.2% ± 2.2%) versus controls (19.2% ± 6.8%; all *p* < 0.01). B‐cell–platelet aggregates were diminished in aICH (49.6% ± 12.0%, decreasing) and CCD (70.3% ± 10.6%, increasing) compared with controls (60.8% ± 14.4%; both *p* < 0.01), but were similar in sICH (57.9% ± 18.3%, *p* = 0.66). The detailed data are shown in Table 3 and Figure 3.

**TABLE 3:** Total study results including leukocyte–platelet aggregation.

	Healthy control (*N* = 48)	aICH (*N* = 20)	sICH (*N* = 30)	CCD (*N* = 20)	*p*		
					aICH vs. control	sICH vs. control	CCD vs. control
Myeloid‐platelet (%)	28.4 ± 8.1	21.1 ± 11.2	26.3 ± 10.5	26.4 ± 9.0	< 0.01**↓	0.17	0.17
Lymphocyte‐platelet (%)	23.2 ± 6.1	11.3 ± 4.7	13.5 ± 5.8	13.9 ± 5.3	< 0.01**↓	< 0.01**↓	< 0.01**↓
Monocyte‐platelet (%)	49.9 ± 14.8	42.3 ± 23.0	52.2 ± 19.7	56.5 ± 15.3	0.15	0.59	0.11
T cells (CD3+, %)	83.7 ± 4.2	83.6 ± 7.5	87.0 ± 6.2	88.5 ± 4.8	0.73	< 0.01**↑	< 0.01**↑
B cells (CD19+, %)	16.3 ± 4.2	16.4 ± 7.5	13.0 ± 6.2	11.5 ± 4.8	0.73	< 0.01**↓	< 0.01**↓
T lymphocyte‐platelet (%)	19.2 ± 6.8	8.9 ± 2.9	13.2 ± 5.1	14.2 ± 2.2	< 0.01**↓	< 0.01**↓	< 0.01**↓
B lymphocyte‐platelet (%)	60.8 ± 14.4	49.6 ± 12.0	57.9 ± 18.3	70.3 ± 10.6	< 0.01**↓	0.66	< 0.01**↑

*Note:* Mean ± SD; *p*‐value by Mann–Whitney test, non‐parametric test.

Abbreviations: aICH, acute intracerebral hemorrhage; CCD, chronic cerebrovascular disease; sICH, subacute intracerebral hemorrhage.

** *p* < 0.01.

## 4 Discussion

The present study demonstrates that patients with intracerebral hemorrhage (ICH) and chronic cerebrovascular disease (CCD) exhibit distinct, stage‐dependent patterns of platelet activation, characterized by differential expression of PAC‐1 and P‐selectin, as well as altered platelet–leukocyte aggregation profiles. Patients with CCD showed persistently impaired α‐granule secretion, evidenced by reduced P‐selectin expression even after stimulation with ADP or TRAP agonists. This chronic suppression suggests long‐term platelet dysfunction associated with sustained vascular and inflammatory stress. In parallel, lymphocyte–platelet aggregates—particularly T‐cell–platelet interactions—were consistently diminished across all patient groups. These findings indicate that platelet–immune communication is dynamically altered following cerebrovascular injury and may remain dysregulated throughout chronic neurovascular disease.

Platelets, once viewed solely as mediators of hemostasis, are now recognized as multifunctional cells linking vascular, immune, and neural systems. Through diverse receptors and signaling pathways, they adapt to inflammatory and neurovascular stress. Structural changes observed in metabolic and neurodegenerative diseases highlight their role as peripheral indicators of brain health and neuroimmune communication. Recent insights emphasize that platelets are not passive fragments but transcriptionally and translationally competent cells capable of synthesizing proteins from pre‐existing mRNAs [24]. This molecular allows platelets to respond rapidly to environmental stimuli and modulate their secretory profile accordingly. Moreover, platelets can endocytose plasma proteins such as fibrinogen and albumin, further expanding their regulatory potential [25]. Collectively, these properties position platelets as dynamic effectors and biosensors linking systemic inflammation, vascular integrity, and neural function.

Under agonist stimulation (e.g., ADP or TRAP), the reduced PAC‐1 binding observed in acute and subacute phase of brain hemorrhage indicates blunted conversion of GPIIb/IIIa (αIIbβ3) to its high‐affinity conformation. This pattern is consistent with defective inside‐out signaling resulting in insufficient integrin activation despite receptor engagement. These findings are consistent with previous observations that acute neurovascular injury and systemic inflammation may lead to transient platelet exhaustion or receptor desensitization [12, 13, 14]. In contrast, CCD patients exhibited increased PAC‐1 positivity, indicating a state of chronic platelet priming or compensatory hyperreactivity. Such upregulation may reflect persistent low‐grade endothelial activation and oxidative stress commonly observed in chronic cerebrovascular disorders [26]. Sustained platelet stimulation under these conditions can lead to continuous turnover and remodeling of platelet receptor expression, which might explain the heightened integrin responsiveness observed in the chronic stage [27]. On the other hand, P‐selectin (CD62P) reflects α‐granule secretion and the externalization of inflammatory mediators. In our cohort, P‐selectin expression was markedly decreased in CCD under both ADP and TRAP stimulation, whereas it was partially preserved in ICH groups under TRAP stimulation. This finding suggests that while integrin activation can recover or even overcompensate over time, granule secretion remains chronically impaired. The persistence of low P‐selectin expression, even in the chronic phase, may indicate depletion of α‐granule stores, defective vesicle trafficking, or long‐term desensitization of the secretory machinery [28]. Previous studies suggest that mitochondrial dysfunction, increased reactive oxygen species, and calcium signaling abnormalities may contribute to this refractory state; however, further evidence is needed [29].

Previous studies have suggested that platelet activation markers may serve as biomarkers in ischemic stroke and cerebral small vessel disease. Kuriyama et al. reported that increased CD62P correlates with white‐matter lesions and cognitive decline, whereas Qiu et al. showed that residual platelet reactivity measured by flow cytometry (including PAC‐1 and CD62P) predicts outcomes in ischemic stroke patients receiving clopidogrel [30, 31]. In contrast, we observed suppressed P‐selectin in CCD, which may reflect late‐stage platelet exhaustion rather than early hyperactivation. Differences across studies likely relate to disease phase, medication exposure, and stimulation protocols. Overall, our findings support a biphasic pattern—suppressed integrin activation in acute/subacute ICH and persistent secretory dysfunction in chronic cerebrovascular disease—with PAC‐1 (activated αIIbβ3) significantly reduced in aICH and sICH.

Beyond hemostasis, platelets actively coordinate immune responses through pattern‐recognition receptors and adhesion molecules (e.g., P‐selectin, CD40L, and integrins) that mediate interactions with leukocytes and endothelial cells, and through secretion of cytokines and chemokines that shape innate and adaptive immunity [32, 33]. In our cohort, platelet–immune aggregates were globally reduced, consistent with platelet exhaustion and/or receptor shedding that may limit sustained immune signaling after hemorrhagic brain injury, although partial recovery of platelet–B‐cell interactions in subacute ICH suggests some restoration during convalescence. Prior experimental work further supports a neuroinflammatory role for platelets: platelet‐derived CD40L promoted microglial activation, astrogliosis, and neuronal death in hypertensive rats, effects that were attenuated by clopidogrel [34]. Collectively, these findings highlight platelets as modulators of neuroinflammation, where prolonged suppression of platelet–lymphocyte interactions may reduce excessive immune activation but could also impede repair mechanisms.

The role of platelets in tissue regeneration has become increasingly apparent. Platelets release growth factors such as PDGF, VEGF, IGF‐1, TGF‐β, and platelet factor 4 (PF4), which promote angiogenesis, neurogenesis, and synaptic remodeling [6, 7]. Apart from hemostasis, inflammation and angiogenesis, another essential aspect of tissue repair following tissue damage is controlled apoptosis [35]. Experimental data indicate that platelet‐derived factors can influence neural stem cell proliferation and survival within neurogenic niches such as the hippocampal dentate gyrus and subventricular zone [36].

In our study, the marked reduction in lymphocyte–platelet, particularly T‐cell–platelet, aggregates suggests disruption of platelet‐mediated adaptive immune regulation after brain injury. Platelets can modulate T‐cell activation and trafficking via direct contact and paracrine signaling, mediated by surface molecules such as P‐selectin, CD40L, ICAM‐2, and GPIbα [37, 38, 39]. During acute ICH, diminished T‐cell–platelet interactions may reflect platelet exhaustion, receptor shedding, or altered activation thresholds under thrombo‐inflammatory stress, potentially exacerbating secondary neuroinflammation and impairing neurovascular repair [40, 41]. Interestingly, the partial recovery of B‐cell–platelet interactions in the chronic phase suggests that platelet–lymphocyte cross‐talk is dynamically regulated rather than uniformly suppressed. However, the persistent deficiency in T‐cell–platelet aggregation may indicate a long‐lasting impairment of platelet‐derived immunoregulatory cues, consistent with the chronic inflammatory phenotype observed in cerebrovascular disease [42]. Overall, these findings in our study support the concept that platelets function as peripheral modulators of neuroimmune homeostasis. By influencing T‐cell activation, cytokine polarization, and migration, platelets may play a crucial role in determining whether post‐injury inflammation is resolved adaptively or progresses toward chronic neurodegeneration.

The limitations of our study include the following: the cross‐sectional design precludes causal inference regarding platelet dysfunction and disease progression. In our cohort, chronic cerebrovascular disease was not entirely attributable to intracranial hemorrhage. Second, medication effects may have confounded platelet reactivity despite our exclusion criteria. Although patients with prior antiplatelet therapy were excluded, other concomitant medications, including anti‐inflammatory agents and antibiotics, may still have influenced platelet function. Third, plasma fibrinogen levels were not measured in this cohort. In plasma‐rich conditions, high fibrinogen may occupy activated αIIbβ3 and partially hinder antibody access, which could reduce measured PAC‐1 binding despite preserved inside‐out activation. Conversely, low fibrinogen could lessen occupancy and potentially increase apparent PAC‐1 binding. Because fibrinogen is the principal ligand for activated αIIbβ3, differences in fibrinogen levels across individuals or groups could potentially affect the apparent PAC‐1 signal through ligand competition or steric effects. Finally, the sample size was modest, and subgroup analyses (e.g., by lesion type or etiology) were limited.

## 5 Conclusion

This study reveals distinct platelet activation signatures across acute and chronic cerebrovascular conditions. Acute ICH is characterized by suppressed integrin activation and diminished platelet–immune aggregation, whereas CCD exhibits persistent α‐granule dysfunction and reduced P‐selectin expression even in the chronic phase. These findings support that platelet dysfunction extends beyond hemostasis to encompass immune dysregulation and neurovascular communication. Flow cytometric profiling of PAC‐1, P‐selectin, and platelet–leukocyte aggregates provides a valuable platform for assessing platelet contributions to brain injury and recovery. Understanding the molecular and temporal dynamics of platelet behavior may open new avenues for diagnostic and therapeutic strategies targeting neurovascular and neuroinflammatory disorders.

## Author Contributions

Chih‐Lung Shen and Li‐Yu Huang were involved in formal analysis and methodology. Jhong‐Kuei Chen and Sheng‐Tzung Tsai were involved in patient enrollment and data collection. Sheng‐Tzung Tsai was also involved in supervision. Yi‐feng Wu was involved in writing the original draft, study design, and supervision.

## Funding

This study was supported by grants TCMMP112‐01‐03, TCMF‐EP 110‐02, and TCMF‐MP 113‐01‐02 from the Buddhist Tzu Chi Medical Foundation, and TCMF‐CM2‐112‐05 from the Hualien Tzu Chi Hospital, Buddhist Tzu Chi Medical Foundation, Hualien, Taiwan.

## Ethics Statement

This study was approved by the Institutional Review Board of Tzu Chi General Hospital (IRB112‐027‐B, IRB109‐049‐B and IRB108‐200‐B).

## Consent

Informed consent was obtained after explaining the study protocol.

## Conflicts of Interest

The authors declare no conflicts of interest.

## Supporting information


**Figure S1:** The gating strategy was designed to identify platelet–leukocyte aggregates associated with specific leukocyte subtypes.
**Figure S2:** The gating strategy was designed to identify platelet–lymphocyte aggregates associated with specific subtypes.

## Data Availability

Due to the IRB, raw data were generated at Hualien Tzu Chi Hospital. Derived data supporting the findings of this study are available from the corresponding author Yi‐Feng Wu on request. Requests to access these datasets should be directed to Yi‐Feng Wu, wuyifeng43@gmail.com.
