# Correction to “Flow Cytometric Profiling Reveals Platelet Dysfunction and Impaired Immune Communication in Acute and Chronic Cerebrovascular Disease”

**DOI:** 10.1002/jcla.70246

**Published:** 2026-04-20

C.‐L. Shen, L.‐Y. Huang, J.‐K. Chen, S.‐T. Tsai, and Y.F. Wu, “Flow Cytometric Profiling Reveals Platelet Dysfunction and Impaired Immune Communication in Acute and Chronic Cerebrovascular Disease” Journal of Clinical Laboratory Analysis (2026): e70192, https://onlinelibrary.wiley.com/doi/10.1002/jcla.70192.

In the “Materials and Methods” section, the text “This study was approved by the Institutional Review Board of Tzu Chi General Hospital (IRB112‐027‐B, IRB109‐049‐B and IRB108‐200‐B).” was incorrect.

The corrected text should read as below:

This study was approved by the Institutional Review Board of Tzu Chi General Hospital (IRB112‐027‐B, IRB109‐049‐B, IRB108‐200‐B, and IRB111‐148‐B)

We apologize for this error.

