# Supplementary material for: Flow Cytometric Profiling Reveals Platelet Dysfunction and Impaired Immune Communication in Acute and Chronic Cerebrovascular Disease
Source: J Clin Lab Anal. 2026 Mar 9;40(7):e70192. doi: 10.1002/jcla.70192 (PMC13052230; doi:10.1002/jcla.70192)
Supplement: Supplementary file 1 — Figure S1: The gating strategy was designed to identify platelet–leukocyte aggregates associated with specific leukocyte subtypes. Figure S2: The gating strategy was designed to identify platelet–lymphocyte aggregates associated with specific subtypes. [file JCLA-40-e70192-s001.docx]

**Supplementary Material for Review**


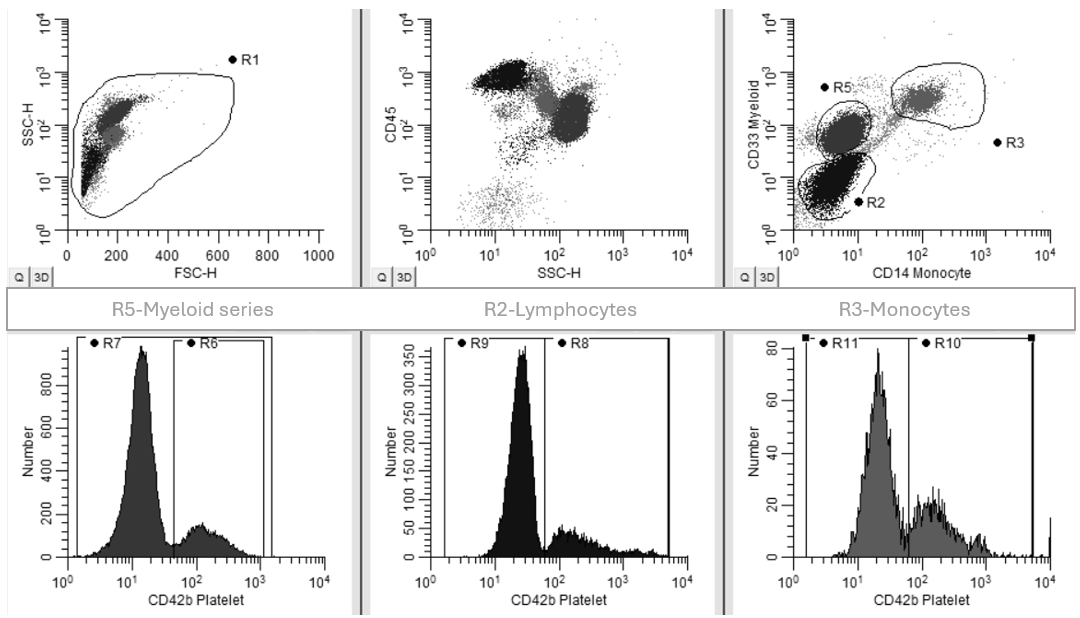


**Figure S1.** The gating strategy was designed to identify platelet–leukocyte aggregates associated with specific leukocyte subtypes. After excluding debris on FSC/SSC plots, leukocytes were gated as CD45-positive events. Within the CD45+ singlet population, myeloid cells were defined by CD33 positivity (including neutrophil-rich events characterized by higher SSC when applicable), and monocytes were identified as CD14-positive events. Platelet–leukocyte aggregates were defined as CD42b-positive events within each leukocyte subset and were quantified as the percentage of CD42b+ events divided by total events in the corresponding parent leukocyte gate.


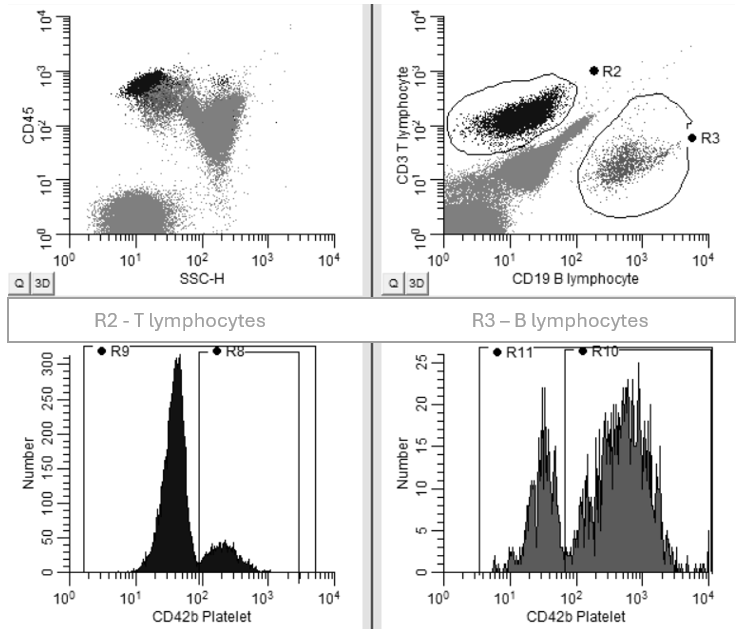


**Figure S2.** The gating strategy was designed to identify platelet–lymphocyte aggregates associated with specific subtypes. T and B lymphocytes were further identified within the lymphocyte gate as CD3-positive and CD19-positive populations, respectively. Platelet–leukocyte aggregates were defined as CD42b-positive events within each leukocyte subset and were quantified as the percentage of CD42b+ events divided by total events in the corresponding parent leukocyte gate.
